# “Impact of stature on non-communicable diseases: evidence based on Bangladesh Demographic and Health Survey, 2011 data”

**DOI:** 10.1186/1471-2458-14-1007

**Published:** 2014-09-26

**Authors:** Md Erfanul Hoque, Mahfuzur Rahman Khokan, Wasimul Bari

**Affiliations:** Department of Statistics, Biostatistics & Informatics, University of Dhaka, Dhaka, 1000 Bangladesh

**Keywords:** Blood glucose level, Blood pressure, Diabetes, Hypertension, Stature

## Abstract

**Background:**

In this paper, an attempt has been made to explore the relationship between height and occurrence of the non-communicable diseases such as diabetes and hypertension.

**Methods:**

For the purpose of analysis, Bangladesh Demographic and Health Survey (BDHS), 2011 data was used. Bivariate analysis along with a Chi-square test was performed to examine association between height and diseases. To measure the impact of stature on diabetes and hypertension, three different logistic regression models (Model I: considering only quartiles of height, Model II: covariates of model I along with demographic variables and Model III: covariates of model II along with clinical variable) were considered.

**Results:**

Occurrence of diabetes and hypertension was found to be inversely related with the height of participants. This inverse association was statistically significant for all three models. After controlling the demographic and clinical variables simultaneously, the odds ratio for highest quartile compared to the lowest quartile was 0.82 with 95% confidence interval (0.69, 0.98) for diabetes; whereas it was 0.72 with 95% confidence interval (0.55, 0.95) for hypertension.

**Conclusions:**

Findings of this paper indicate that persons with shorter stature are substantially more likely to develop diabetes as well as hypertension. The occurrence of non-communicable diseases like diabetes and hypertension can be reduced by controlling genetic and non-genetic (early-life and childhood) factors that may influence the height.

## Background

A rapid increase of Non-communicable diseases (NCDs) is becoming an alarming threat for the achievement of global progress in the developed and developing countries. The NCDs kill more than 36 million people each year, among them 80% of NCD death occur in low- and middle-income countries like Bangladesh [1]. This death toll will rise unless proper measures are taken. If the current trend continues, by 2020, NCDs will be responsible for 73% of total death and 60% of disease burden in the developing countries [1]. The causal risk factors of NCD risk factors operate through intermediate risk factors such as high blood pressure, elevated blood glucose level and plasma lipid levels [2]. These are the most prevalent risk factors around the world [3]. Generally, these risk factors are modifiable and preventable. Therefore, early identification and preventive behavior for these risk factors can reduce the risk of developing cardiovascular disease by 80% and risk of type II diabetes by 90% [4]. Like other developing countries, Bangladesh is making a transition in the disease and death patterns from communicable diseases to NCDs [5, 6]. The prevention and control NCDs depend on the availability of information about these diseases and the intermediate biological biomarkers. The 2011 BDHS is the first national survey to include biomarker measurements for blood pressure and fasting blood glucose. In this paper, an attempt has been made to identify potential factors associated with the occurrence of diabetes, which is based on blood glucose level and hypertension, which is based on blood pressure.

The association between height of respondent and diabetes risk has been investigated by several epidemiological studies but it is still unclear whether height is associated as a risk factor. Height appears to be inversely related with diabetes, however there have been contravening reports about possible association of heights and diabetes [7–11]: a positive association was found in a study [10], whereas no association [12] or an inverse relation was reported in others [8, 11]. Also, there was an association only in women [9] or men [13]. Hence, it would be interesting to find out a relationship between height and development of diabetes. In this paper, we try to investigate this relationship in the context of Bangladesh using BDHS 2011 data.

In a sample survey of non-communicable disease in an urban African adult population [14] and in some other studies [15–18], it is found that there is no association between height and hypertension risk. It is also found that short stature is associated with higher risk of cardiovascular heart disease [19–22]. A U-shaped association is found with quartiles of stature, mainly among women [23]. The leg length has recently been identified as a risk factor for the cardiovascular disease [24]. After adjusting age, gender, socioeconomic status and life style habits, longer legs were found to be associated with lower pulse pressure and lower low-density lipoprotein cholesterol [25]. In this paper, an attempt has also been made to examine the association between the height and hypertension. The potential factors that affect diabetes and hypertension are determined as well.

## Methods

### Database

The study used the 2011 Bangladesh Demographic and Health Survey (BDHS), a nationally representative sample survey of men and women of reproductive age that provides information on childhood mortality levels, fertility preferences, use of family planning methods, and maternal, child, and newborn health. Provision of biomarker indices of adult male and female populations is the special characteristic of this survey, which are instrumental in determining the increasing risk of NCDs. In the 2011 BDHS survey, a subsample of women and men age 35 years and older were eligible to participate in the biomarker component, which included blood pressure measurements, blood glucose testing, and height and weight measurements. There were 4,311 women and 4,524 men age 35 and older for blood pressure and blood glucose measurement. Among these individuals, 92 percent of women and 86 percent of men took part in the blood pressure measurement, and 89 percent of women and 83 percent of men participated in the blood glucose measure measurement.

In this study, the secondary data set ‘Bangladesh Demographic and Health Survey, 2011 has been used. This data set was collected through a collaborative effort of the National Institute of Population Research and Training (NIPORT), ICF International (USA) and Mitra and Associates. The written informed consent was obtained from the participants prior to participation in the study and data collection was conducted confidentially. For this study, there is no need ethical approval since it is based on publically available secondary data.

### Definitions of diseases

The 2011 BDHS uses WHO cut-off points for measuring fasting plasma glucose (FPG) [26]. The cut-off points correspond to the clinical classification for normal FPG levels, pre-diabetes and diabetes. If FPG is between 3.9 mmol/l (70 mg/dl) and 6.0 mmol/l (108 mg/dl), participants are considered to be normal. Participants with FPG value of 6.1-6.9 mmol/l (110–124 mg/dl) are classified as prediabetic, and individuals with FPG greater than or equal to 7.0 mmol/l (126 mg/dl) are considered as diabetic. In this paper, an individual is considered to be diabetic if his/her FPG value is greater than 6.1 mmol/l (110 mg/dl), otherwise he/she is considered to be in normal health state or non-diabetic [2].

The 2011 BDHS uses the American Heart Association guidelines for cut-off points for blood pressure measurements [27]. The cut-off points represent to the clinical classification for hypertension as they relate to the systolic and diastolic blood pressure measurement. For the purpose of analysis, in this paper, we divide individuals into two categories: having hypertension and not having hypertension. An individual is said to have hypertension if his/her systolic blood pressure is greater than 140 mmHg or his/her diastolic blood pressure is greater than 90 mmHg [28].

Along with two outcome variables diabetes and hypertension, we consider height as categorical variable based on quartiles, gender (male, female), education level (no education, primary, secondary, higher), wealth index (poorest, poorer, middle, richer, richest), place of residence (rural, urban), division (Dhaka, Barisal, Chittagong, Khulna, Rajshahi, Rangpur, Shylet), body mass index (BMI) category [thin (BMI < 18.5), normal (BMI 18.5-24.9), overweight (BMI > 24.9)] as determinants of these outcomes [11–13, 25, 28].

To examine the relationship between different determinants and having diabetes and hypertension, first we conduct a bivariate analysis along with a Chi-square test and then we examine the effects of covariates that are found to be statistically significant in the bivariate analysis by fitting logistic regression model [29]. Since the main concern of this paper is to find a relationship between height and having diabetes and hypertension, at the stage of regression analysis, we consider three logistic regression models. These are Model 1: considering only the quartiles of height to examine the gross effect of the height; Model II: covariate of Model I along with demographic variables (education level, wealth index, place of residence, division) to examine the effect of height after controlling the demographic variables; and Model III: covariates in Model II along with clinical variable (BMI) to examine the effect of height after controlling the demographic as well as the clinical variables. For the purpose analysis, the SPSS for Windows (SPSS Inc., Chicago, IL, USA) is used.

## Results

### Univariate analysis

The mean height of respondent is found to be 1.57 meter with standard deviation 0.091 meter. The minimum and maximum heights are 1.09 and 1.87 meters, respectively. Among the participants, 51.21% are male and 48.79% are female. It is observed that 34.6% of participants have no education and 32.4%, 24.2%, and 8.8% of respondents have primary, secondary and higher education, respectively. Most of the respondents (66.43%) are found to reside in the rural area. The distribution of respondents among the divisions Dhaka, Barisal, Chittagong, Khulna, Rajshahi, Rangpur, Shylet are 17.12%, 11.71%, 17.11%, 13.53%, 13.46%, 13.33%, and 13.74%, respectively. More than half of the participants (57.45%) are in the normal category of BMI measurement scale. It is found that 30.49% are thin and 12.06% are overweighed. Around 20% of respondents fall in the each category of wealth index. One-third of respondents (33.3%) are found to be diabetic and 21.4% of respondents have hypertension.

### Bivariate analysis

#### Diabetes

The background characteristics of the study participants by diabetes status are shown in Table 1. It is interesting to observe that prevalence of diabetes increase as height of participant decreases. Individuals who belong to the lower quartile of height are more likely to be diabetic compared to their counterparts. The percentage of diabetes is almost equal among men (33.0%) and women (33.6%). Considering education level it can be mentioned that if education level increases the percentage of diabetes also increases. Among the wealth index, the richest group has higher proportion of diabetics (40.4%) whereas the other groups are more or less similar (around 31%). Prevalence of diabetes is more in urban area (35.4%) compared to the rural area (32.2%). Among the divisions, participants from Chittagong are most likely to have diabetes (42.0%), whereas this is least in the Rangpur division (28.0%). It is observed that overweighed and thin participants are more prone to be diabetic than normal weighed participants. Among the overweighed participants, the rate of occurrence of diabetes is 50.5%, whereas it is 33.6% and 30.6% for the thin and normal categories of BMI, respectively. Note that height of participants is significantly associated with the occurrence of diabetes at 5% level of significance. The associations between diabetes levels and education level, wealth index, place of residence, division and BMI category are found to be highly significant (at 1% level of significance). Only gender of participant shows insignificant association with levels of diabetes.

**Table 1:** **Association between selected variables and the occurrence of diabetes with** ***p*** **values**

Characteristics	Diabetes status		***p***value
	Non-diabetes	Diabetes	
	n (%)	n (%)	
**Height category**			
Q1: (≤1.51)	854 (63.8%)	485 (36.2%)	0.037
Q2: (1.51-1.583)	865 (65.7%)	452 (34.3%)	
Q3: (1.583-1.637)	906 (67.8%)	430 (32.2%)	
Q4: (≥1.673)	875 (67.2%)	429 (32.9%)	
**Gender**			
Female	2543 (66.4%)	1288 (33.6%)	0.558
Male	2503 (67.0%)	1231 (33.0%)	
**Education level**			
No education	2384 (69.5%)	1047 (30.5%)	<0.001
Primary	1377 (66.1%)	706 (33.9%)	
Secondary	915 (65.2%)	489 (34.8%)	
Higher	370 (57.2%)	277 (42.8%)	
**Wealth index**			
Poorest	929 (68.9%)	419 (31.1%)	<0.001
Poorer	935 (69.1%)	418 (30.9%)	
Middle	1010 (68.9%)	455 (31.1%)	
Richer	1090 (68.8%)	494 (31.2%)	
Richest	1082 (59.6%)	733 (40.4%)	
**Place of residence**			
Urban	1607 (64.6%)	882 (35.4%)	0.006
Rural	3439 (67.8%)	1637 (32.2%)	
**Division**			
Dhaka	929 (70.6%)	387 (29.4%)	<0.001
Barisal	669 (59.8%)	450 (40.2%)	
Chittagong	502 (58.0%)	364 (42.0%)	
Khulna	889 (73.8%)	316 (26.2%)	
Rajshahi	717 (67.1%)	351 (32.9%)	
Rangpur	769 (72.0%)	299 (28.0%)	
Shylet	571 (61.9%)	352 (38.1%)	
**BMI category**			
Thin	1062 (66.4%)	538 (33.6%)	<0.001
Normal	2075 (69.4%)	915 (30.6%)	
Overweight	310 (49.5%)	316 (50.5%)	

#### Hypertension

The distribution of background characteristics by the levels of hypertension is given in Table 2. Like diabetic patients, occurrence of hypertension is inversely related with the height of patient. The rates of having hypertension in the four quartiles of height are 32.8%, 22.4%, 16.2% and 17.4%, respectively. This association is statistically significant with *p* value <0.001. Unlike diabetes, gender of participant is significantly associated with the levels of hypertension (*p* value <0.001). Female participants are more likely to have hypertension than their counterpart (25.7% versus 17.0%). The pattern of associations between levels of hypertension and education level, wealth index and place of residence are almost similar to the pattern of associations observed for the occurrence of diabetes. The rate of occurrence of hypertension significantly (*p* value <0.001) changes with divisions. Participants from Rangpur are most likely to have hypertension (27.6%), whereas this is least in the Shylet division (16.0%). It is noticed that with the increase of BMI, the occurrence of hypertension is significantly increased (*p* value <0.001).

**Table 2:** **Association between selected variables and the occurrence of hypertension with** ***p*** **values**

Characteristics	Hypertension status		***p***value
	No hypertension	Hypertension	
	n (%)	n (%)	
**Height category**			
Q1: (≤1.51)	935 (67.2%)	456 (32.8%)	<0.001
Q2: (1.51-1.583)	1068 (77.6%)	309 (22.4%)	
Q3: (1.583-1.637)	1168 (83.8%)	225 (16.2%)	
Q4: (≥1.673)	1138 (82.6%)	240 (17.4%)	
**Gender**			
Female	2959 (74.3%)	1022 (25.7%)	<0.001
Male	3243 (83.0%)	663 (17.0%)	
**Education level**			
No education	2786 (77.6%)	804 (22.4%)	0.003
Primary	1760 (81.1%)	411 (18.9%)	
Secondary	1151 (79.1%)	305 (20.9%)	
Higher	505 (75.4%)	165 (24.6%)	
**Wealth index**			
Poorest	1171 (83.5%)	232 (16.5%)	<0.001
Poorer	1171 (82.0%)	257 (18.0%)	
Middle	1243 (81.3%)	286 (18.7%)	
Richer	1267 (77.2%)	375 (22.8%)	
Richest	1350 (71.6%)	535 (28.4%)	
**Place of residence**			
Urban	1940 (74.9%)	649 (25.1%)	<0.001
Rural	4262 (80.4%)	1036 (19.6%)	
**Division**			
Dhaka	1045 (77.5%)	304 (22.5%)	<0.001
Barisal	757 (80.8%)	180 (19.2%)	
Chittagong	992 (83.8%)	192 (16.2%)	
Khulna	911 (73.6%)	327 (26.4%)	
Rajshahi	892 (79.9%)	225 (20.1%)	
Rangpur	794 (72.4%)	302 (27.6%)	
Shylet	811 (84.0%)	155 (16.0%)	
**BMI category**			
Thin	1374 (82.7%)	288 (17.3%)	<0.001
Normal	2444 (78.0%)	690 (22.0%)	
Overweight	441 (67.0%)	217 (33.0%)	

### Regression analysis

#### Diabetes

One of the main purposes of this study is to examine the effect of height of participant on the occurrence of diabetes. For this purpose, we consider three logistic regression models. The results are given in Table 3. In Model I, only height of participants is considered to measure the unadjusted effect of height. It is observed that height is almost inversely related with the development of diabetes. For example, individual who is in the highest quartile of height are 14% less likely to develop diabetes compared to the individual who is in the lowest quartile of height. Odds ratios for the second and third quartiles are found to be statistically significant at 5% and 10% level of significance, respectively. It is interesting to observe from Model II and Model III that the relationship between height and developing diabetes is becoming strictly inversed when demographic and clinical variables are added to the Model I. In Model II and Model III, both odds ratios for the second and third quartiles are significant at 5% level of significance. It implies that height of participant plays an important role in developing the diabetes as it is found statistically significant even after controlling the demographic and clinical variables.

**Table 3:** **Regression coefficients (Reg. Coef.) and odds ratios (OR) with 95% confidence intervals (95% CI) of explanatory variables for the occurrence of diabetes obtained from logistic regression model**

	Model I	Model II	Model III
Characteristics	OR (95% CI)	OR (95% CI)	OR (95% CI)
**Height category**			
Q1: (≤1.51) (Ref)	1.00	1.00	1.00
Q2: (1.51-1.583)	0.92 (0.79, 1.08)	0.92 (0.78, 1.08)	0.93 (0.79, 1.10)
Q3: (1.583-1.637)	0.84 (0.71, 0.98)**	0.81 (0.68, 0.95)**	0.84 (0.71, 1.00)**
Q4: (≥1.673)	0.86 (0.74, 1.01)*	0.79 (0.67, 0.95)**	0.82 (0.69, 0.98)**
**Education level**			
No education (Ref)		1.00	1.00
Primary		1.06 (0.92, 1.23)	1.06 (0.91, 1.23)
Secondary		1.01 (0.84, 1.21)	0.97 (0.81, 1.17)
Higher		1.5 (1.2, 1.94)***	1.39 (1.09, 1.77)**
**Wealth index**			
Poorest		1.12 (0.92, 1.36)	1.09 (0.89, 1.33)
Poorer		1.15 (0.95, 1.39)	1.13 (0.93, 1.37)
Middle (Ref)		1.00	1.00
Richer		1.05 (0.87, 1.27)	1.03 (0.85, 1.25)
Richest		1.6 (1.3, 1.97)***	1.46 (1.18, 1.79)***
**Place of residence**			
Rural (Ref)		1.00	1.00
Urban		1.06 (0.93, 1.23)	1.11 (0.96, 1.29)
**Division**			
Dhaka (Ref)		1.00	1.00
Barisal		1.9 (1.54, 2.38)***	1.91 (1.53, 2.39)***
Chittagong		1.7 (1.37, 2.05)***	1.63 (1.33, 2.00)***
Khulna		0.85 (0.69, 1.05)	0.83 (0.67, 1.03)*
Rajshahi		1.24 (1.0, 1.54)***	1.19 (0.96, 1.49)
Rangpur		0.98 (0.79, 1.21)	0.95 (0.77, 1.18)
Shylet		1.62 (1.31, 2.01)**	1.59 (1.28, 1.97)***
**BMI category**			
Thin			1.17 (1.03, 1.34)**
Normal (Ref)			1.00
Overweight			2.09 (1.74, 2.52)***

*Notes.*

***p value < 0.001, **p value < 0.05, *p value < 0.10.

It is found from Model II that rate of occurring diabetes increases significantly with level of education and wealth index. For individual with highest education level, the occurrence of diabetes is 50% higher than the individual with no education. Those who are in the highest wealth index level are 60% more likely to have diabetes than who are in the middle level. Place of residence is not found to have statically significant effect, but regions have significant effects on diabetes. Similar results are found in the model when clinical variable BMI is added to Model II. Both thin and overweighed individuals are positively associated with the development of diabetes than their counterpart normal weighed. These effects are statistically significant at 5% and 1% level of significance, respectively.

#### Hypertension

To determine the potential factors associated with the occurrence of hypertension, following the analysis for diabetes, three logistic regression models are fitted and the results are given in Table 4. The unadjusted effect of height on the hypertension is statistically significant for all three quartiles (second, third, and fourth). Like in diabetes, it is also found that height of participant is almost inversely related with the development of hypertension. This relation remains same in the other two models (in Model II and Model III) after adjusting for demographic and clinical variables. In Model I, the odds ratio (OR) for the highest quartile is found to be 0.43 with 95% confidence interval (CI) (0.36, 0.52) and this effect is statistically significant at 1% level of significance. The prevalence of hypertension is decreased by 57% for those individuals who belong to the highest quartile of height compared to individuals in the lowest quartile. This OR is 0.74 with 95% CI (0.57, 0.97) in the Model II; whereas it is 0.72 in the Model III with 95% CI (0.55, 0.95). It indicates that as the participant is taller, lower would be the rate of developing the hypertension. These results establish the fact that short stature is a potential risk factor for developing hypertension.

**Table 4:** **Regression coefficients (Reg. Coef.) and odds ratios (OR) with 95% confidence intervals (95% CI) of explanatory variables for the occurrence of hypertension obtained from logistic regression model**

	Model I	Model II	Model III
Characteristics	OR (95% CI)	OR (95% CI)	OR (95% CI)
**Height category**			
Q1: (≤1.51) (Ref)	1.00	1.00	1.00
Q2: (1.51-1.583)	0.59 (0.50, 0.70)***	0.89 (0.72, 1.08)	0.88 (0.72, 1.08)
Q3: (1.583-1.637)	0.39 (0.33, 0.47)***	0.71 (0.55, 0.92)**	0.69 (0.53, 0.90)***
Q4: (≥1.673)	0.43 (0.36, 0.52)***	0.74 (0.57, 0.97)**	0.72 (0.55, 0.95)**
**Gender**			
Male (Ref)		1.00	1.00
Female		0.44 (0.36, 0.54)***	0.45 (0.36, 0.56)***
**Education level**			
No education (Ref)		1.00	1.00
Primary		0.90 (0.76, 1.07)	0.87 (0.73, 1.04)
Secondary		0.96 (0.78, 1.19)	0.92 (0.75, 1.15)
Higher		1.26 (0.96, 1.65)*	1.14 (0.87, 1.50)
**Wealth index**			
Poorest		0.77 (0.61, 0.97)**	0.78 (0.62, 0.99)**
Poorer		0.84 (0.67, 1.05)	0.86 (0.68, 1.08)
Middle (Ref)		1.00	1.00
Richer		1.19 (0.97, 1.47)	1.14 (0.92, 1.41)
Richest		1.63 (1.3, 2.04)***	1.5 (1.19, 1.9)***
**Place of residence**			
Rural (Ref)		1.00	1.00
Urban		0.92 (0.79, 1.08)	0.94 (0.80, 1.11)
**Division**			
Dhaka (Ref)		1.00	1.00
Barisal		0.82(0.63,1.06)	0.84 (0.65, 1.1)
Chittagong		0.67(0.52, 0.85)***	0.67 (0.52, 0.86)***
Khulna		1.44(1.15, 1.79)***	1.45 (1.16, 1.81)***
Rajshahi		0.97(0.77, 1.24)	0.98 (0.77, 1.26)
Rangpur		1.45 (1.15,1.82)***	1.48 (1.73, 1.87)***
Shylet		0.71 (0.55, 0.92)	0.73 (0.56, 0.95)**
**BMI category**			
Thin			0.72 (0.61, 0.85)***
Normal (Ref)			1.00
Overweight			1.33 (1.1, 1.63)***

*Notes.*

***p value < 0.001, **p value < 0.05, *p value < 0.10.

In Model II, the effects of demographic variables on the occurrence of hypertension are also examined along with the effect of height of respondents. It is observed that female is less likely to have hypertension compared to male. In this case, the OR is found to be 0.44 with 95% CI (0.36, 0.54). Only higher education level is found to have significant impact on the hypertension. The odds of developing hypertension is 26% higher for an individual with higher education compared to an individual with no education. It is interesting to observe that the development of hypertension increases with the level of wealth index. For example, ORs are 0.77 [95% CI: (0.61, 0.97)] and 1.63 [95% CI: (1.30, 2.04)] for the poorest and richest groups, respectively. Like in the case of diabetes, place of residence of respond is found to have no significant effect on the hypertension. Individuals from Rangpur and Khulna divisions are more prone to the hypertension compared to individuals from other divisions. In Model III, effect of BMI is examined controlling demographic variables as well as height of individuals. The pattern of effects of demographic variables in Model III is same as the pattern found in Model II. There exists a positive linear relationship between the levels of BMI and occurrence of hypertension. Thin is less likely [OR: 0.72; 95% CI: (0.61, 0.85)] and overweighed is more likely to have hypertension compared to the individual with normal weight.

## Discussion and conclusions

The aim of this study is to find out the relationship between height of respondent and the occurrence of diabetes and hypertension. For this purpose, we conduct, first, bivariate analysis and then regression analysis by fitting three different logistic regression models. In Model I, only the quartiles of height on individual is considered to examine the unadjusted effect of height on diabetes and hypertension. Effect of height is also examined in Model II by controlling demographic variables and in Model III by controlling demographic and clinical variables. From both bivariate analysis and all three logistic regression models, it is found that occurrence of diabetes and hypertension is inversely related with the height of participants and this association is statistically significant. That is, chance of developing diabetes and hypertension decreases with the increase of height of participant.

It is not clear how height is inversely related with the occurrence of diabetes. One of explanations of this inverse relation is that taller individuals have more muscle mass and muscle is the major tissue involved in uptake of glucose, against the fixed glucose load of 75 grams [7]. The dilution effect of total body water may contribute in establishing the results [7]. The possible explanations for the inverse relationship between height and the occurrence of hypertension were discussed by Lawlor et al. [24]. These are: genetic factors determining growth patterns may be associated with hypertension [30]; coronary artery vessel diameter increases with height and vessel with smaller diameters may result in clinical disease outcome with relatively smaller amounts of atherosclerosis.

Besides height of respondents, regression analyses of large databases have shown that education level, wealth index, place of residence, division, and body mass index are associated with the occurrence of diabetes. Along with the factors associated with diabetes, gender is also associated with the occurrence of hypertension.

One of the strengths of this study is the use of a nationwide large sample with comprehensive information on the occurrence of diabetes and hypertension, anthropometric and demographic variables. Anthropometric variables are collected by using direct measurement rather than self-reporting. This data set is collected through a reliable and uniform procedure, which minimize the measurement error and bias. The response rates of this study are high.

The main limitation of this paper is to use a cross-sectional study and hence it may produce selection and information bias. Moreover, from this study, it may not be possible to assess the changes in the association between height and occurrence of diabetes as well as hypertension over the time. In our It suggests that shorter individuals may be at higher risk of metabolic disturbance [31]. Controlling factors which may have an influence on height could therefore result in a reduction of NCDs such as diabetes and hypertension. study, we only consider individuals of age 35 years or more since in 2011 BDHS study, men and women of age 35 or older were only considered to provide the biomarker information. Therefore, results of this study may not be extended to the other age groups. Though the variables on life styles such as diet, physical exercise, and smoking are the potential confounders for the diabetes and hypertension, these were not included in the analysis since these are not available in the 2011 BDHS data.

The result of this study reveals the fact that there exists an inverse relationship between height and the occurrence of diabetes as well as hypertension. That is, the shorter height is associated with a higher occurrence of diabetes as well as hypertension. The height may be controlled by genetic and non-genetic (early-life and childhood) factors [32–34]. Naturally, if most of the members of a family are of short stature, the next generation is likely to have short stature. But, genetic factors are entirely beyond the control of human. The non-genetic factors which may affect the height include: maternal smoking during pregnancy, prenatal and postnatal, ill health during childhood and adolescence, birth weight, mental condition during childhood and adolescence. Non-genetic factors can be controlled to some extent by following a healthy life style from the childhood.

In future studies, a cohort should be followed with longitudinal data collected at an early age prior to development of illness to establish the relationship between the stature of an individual and occurrence of non-communicable disease. One may also take the genetic and non-genetic factors of stature into account for further analysis.

## Abbreviations

BDHS: Bangladesh Demographic and Health Survey
FPG: Fasting plasma glucose
NCD: Non-communicable disease
WHO: World Health Organization.
